# Controllability and Observability of Fractional Linear Systems with Two Different Orders

**DOI:** 10.1155/2014/618162

**Published:** 2014-01-20

**Authors:** Dengguo Xu, Yanmei Li, Weifeng Zhou

**Affiliations:** Department of Mathematics, Chuxiong Normal University, Chuxiong, Yunnan 675000, China

## Abstract

This paper is concerned with the controllability and observability for a class of fractional linear systems with two different orders. The sufficient and necessary conditions for state controllability and state observability of such systems are established. The results obtained extend some existing results of controllability and observability for fractional dynamical systems.

## 1. Introduction

In the last three decades, interest in fractional calculus has experienced rapid growth and at present we can find many papers devoted to its theoretical and application aspects; see the work of [1] and the references therein. Fractional order models of real systems are often more adequate than the usually used integer order models in electrochemistry [2], advection dispersion models [3], anomalous diffusion [4], viscoelastic materials [5], fractal networks [6–8], robotics [9], and so forth. Further, during recent years a renewed interest has been devoted to fractional order systems in the area of automatic control; the reader can refer to monograph [10]. Oustaloup [11] initiated the first framework for noninteger order systems in the automatic control area. Fractional order control is the use of fractional calculus in the aforementioned topics, the system being modeled in a classical way or as a fractional one. From a certain point of view, the applications of fractional calculus have experienced an evolution analogous to that of control following two parallel paths depending on the starting point: the time domain or the frequency domain [12–14].

Controllability and observability are two of the most fundamental concepts in modern control theory. They have close connections to pole assignment, structural decomposition, quadratic optimal control, observer design, and so forth [15, 16]. In the past ten years, many results have been obtained on controllability and observability of fractional order systems. Chen et al. [17] proposed robust controllability for interval fractional order linear time invariant systems, whereas Adams and Hartley [18] studied finite time controllability for fractional systems. The controllability conditions for fractional control systems with control delay were obtained in [19]. Shamardan and Moubarak [20] extended some basic results on the controllability and observability of linear discrete-time fractional order systems and developed some new concepts inherent to fractional order systems with analytical methods for checking their properties. Balachandran et al. [21] obtained controllability criteria for fractional linear systems, and then this result is extended to nonlinear fractional dynamical systems by using fixed point theorem. In recent paper [22], necessary and sufficient conditions of controllability and observability for fractional linear time invariant system are included.

However, to the best of our knowledge, there has been no result about the controllability and observability of fractional linear systems with different orders. In this paper, we investigated state controllability and state observability of fractional linear systems with two different orders. We derive the sufficient and necessary conditions on controllability and observability for the fractional linear systems with two different orders.

The paper is organized as follows.  Section 2 formulates the problem and presents the preliminary results. The main results about controllability and observability for the fractional linear systems with two different orders are given in Sections 3 and 4, respectively. Finally, some conclusions are drawn in Section 5.

## 2. Preliminaries

Consider the following fractional linear systems with two different orders:

(1) $$\begin{array}{c} \left[\begin{array}{c} {}^{c}D_{t}^{\alpha }x_{1}\left(t\right)\\ {}^{c}D_{t}^{\beta }x_{2}\left(t\right) \end{array}\right]=\left[\begin{array}{cc} A_{11} & A_{12}\\ A_{21} & A_{22} \end{array}\right]\left[\begin{array}{c} x_{1}\left(t\right)\\ x_{2}\left(t\right) \end{array}\right]+\left[\begin{array}{c} B_{1}\\ B_{2} \end{array}\right]u\left(t\right), \end{array}$$

where ^*c*^ *D*_*t*_^*α*^, ^*c*^ *D*_*t*_^*β*^ are the Caputo derivative, 0 < *α* < 1, 0 < *β* < 1; *x*_1_ ∈ *R* ^*n*_1_^, *x*_2_ ∈ *R* ^*n*_2_^ are the state vectors; *A*_*ij*_ ∈ *R* ^*n*_*i*_×*n*_*j*_^, *B*_*i*_ ∈ *R* ^*n*_*i*_×*m*^,  *i*, *j* = 1,2, are the known constant matrices; *n*_1_ + *n*_2_ = *n*; *u* ∈ *R* ^*m*^ is the input vector.

When *A*_12_ = *A*_21_ = 0, the system (1) reduces to the following form:

(2) $$\begin{array}{c} \left[\begin{array}{c} {}^{c}D_{t}^{\alpha }x_{1}\left(t\right)\\ {}^{c}D_{t}^{\beta }x_{2}\left(t\right) \end{array}\right]=\left[\begin{array}{cc} A_{11} & 0\\ 0 & A_{22} \end{array}\right]\left[\begin{array}{c} x_{1}\left(t\right)\\ x_{2}\left(t\right) \end{array}\right]+\left[\begin{array}{c} B_{1}\\ B_{2} \end{array}\right]u\left(t\right). \end{array}$$

We first give some definitions about fractional calculus; for more details, see [10, 23, 24].

**Definition 1**

Riemann-Liouville's fractional integral of order *α*  (*α* > 0) for a function *h* : (0, *∞*) → *R* is defined as

(3) $$\begin{array}{c} {\;}_{0}D_{t}^{-\alpha }h\left(t\right)=\frac{1}{\Gamma \left(\alpha \right)}\int_{0}^{t}{\left(t-s\right)}^{\alpha -1}h\left(s\right)ds, \end{array}$$

where Γ(*α*) = ∫_0_^*∞*^ *t* ^*α*−1^ *e* ^−*t*^ *dt* is Gamma function.

**Definition 2**

Riemann-Liouville's fractional derivative of order *α*  (0 < *α* < 1) for a function *h* : (0, *∞*) → *R* is defined as

(4) $$\begin{array}{c} {\;}_{0}D_{t}^{\alpha }h\left(t\right)=\frac{1}{\Gamma \left(1-\alpha \right)}\frac{d}{dt}\int_{0}^{t}{\left(t-s\right)}^{-\alpha }h\left(s\right)ds. \end{array}$$

**Definition 3**

The Caputo fractional derivative of order *α*  (0 < *α* < 1) for a function *h* : (0, *∞*) → *R* is defined as

(5) $$\begin{array}{c} {\;}_{0}^{c}D_{t}^{\alpha }h\left(t\right)=\frac{1}{\Gamma \left(1-\alpha \right)}\int_{0}^{t}{\left(t-s\right)}^{-\alpha }h^{\prime }\left(s\right)ds. \end{array}$$

Throughout the paper, only the Caputo definition is used since the Laplace transform allows using initial values of classical integer order derivatives with clear physical interpretations.

According to [25], the solution of the system (1) can be obtained. Therefore, the following lemma holds.

**Lemma 4**

The solution of system (1) with initial conditions *x*_1_(0) = *x*_10_ and *x*_2_(0) = *x*_20_ is given by

(6) $$\begin{array}{c} x\left(t\right)=\Phi_{0}\left(t\right)x_{0}+\int_{0}^{t}\Phi \left(t-\tau \right)Bu\left(\tau \right)d\tau , \end{array}$$

where

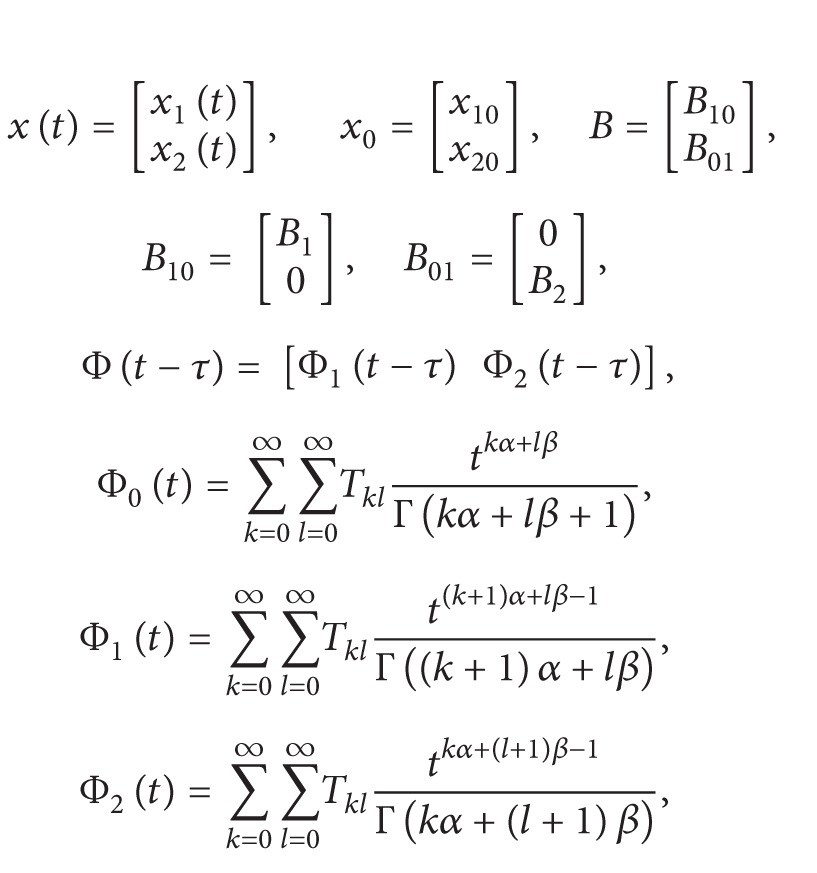
(7)

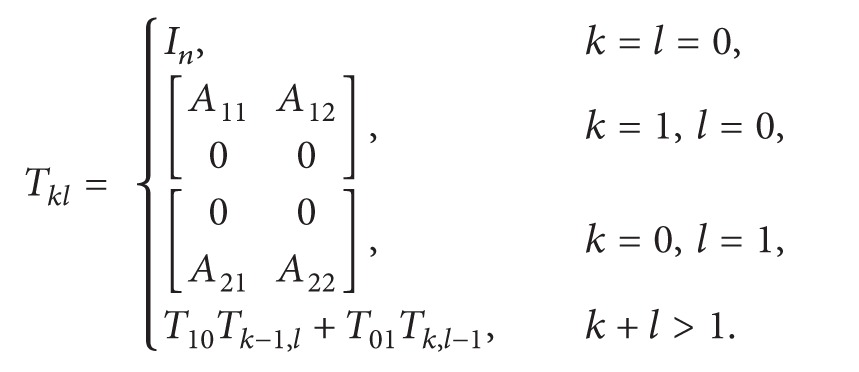
(8)

From (8), the following lemma holds.

**Lemma 5**

The implication

(9) $$\begin{array}{c} \sum_{k+l=m}T_{kl}=A^{m},\;m\in Z^{+}, \end{array}$$

Holds, where

(10) $$\begin{array}{c} A=\left[\begin{array}{cc} A_{11} & A_{12}\\ A_{21} & A_{22} \end{array}\right]. \end{array}$$

**Proof**

When *m* = 1, it follows from (8) that

(11) $$\begin{array}{c} \sum_{k+l=1}T_{kl}=T_{01}+T_{10}=A, \end{array}$$

which implies that (9) holds when *m* = 1. Now, suppose that (9) is true when *m* = *p*,  *p* ∈ *Z* ^+^; namely,

(12) $$\begin{array}{c} \sum_{k+l=p}T_{kl}=A^{p}. \end{array}$$

When *m* = *p* + 1, we get

(13) ∑k+l=p+1Tkl=∑k+l=p+1(T10Tk−1,l+T01Tk,l−1)=∑k+l=p+1T10Tk−1,l+∑k+l=p+1T01Tk,l−1=∑k+l=pT10Tk,l+∑k+l=pT01Tk,l=T10Ap+T01Ap=Ap+1,

which means that (9) holds when *m* = *p* + 1. Reasoning by mathematical induction, we can immediately conclude that (9) is true for any *m* ∈ *Z* ^+^. This therefore completes the proof.

## 3. Controllability

In this section, the sufficient and necessary conditions of controllability for the fractional linear system (1) with two different orders are discussed based on previous definitions and results. Similar to the concepts of controllability for general fractional linear systems, the definition of controllability for fractional linear systems with different orders is given as follows.

**Definition 6**

The system (1) is called state controllable on [0, *T*] if given any state *x*_0_, *x*_*t*_1__ ∈ *R* ^*n*^ there exists a control input signal *u*(*t*) : [0, *T*] → *R* ^*m*^ such that the corresponding solution of system (1) satisfies *x*(0) = *x*_0_ and *x*(*t*_1_) = *x*_*t*_1__,  *t*_1_ ∈ [0, *T*].

**Theorem 7**

The system (1) is controllable on [0, *t*_1_] if and only if the controllability Gramian matrix

(14) $$\begin{array}{c} W_{c}\left(0,t_{1}\right)=\int_{0}^{t_{1}}\Phi \left(t_{1}-\tau \right)BB^{T}\Phi^{T}\left(t_{1}-\tau \right)d\tau \end{array}$$

is nonsingular.

**Proof**

Suppose that the matrix *W*_*c*_(0, *t*_1_) is nonsingular. Accordingly, *W*_*c*_(0, *t*_1_) is invertible. Then given an initial state *x*(0) = *x*_0_ ≠ 0, choose

(15) $$\begin{array}{c} u\left(t\right)=B^{T}\Phi^{T}\left(t_{1}-t\right)W_{c}^{-1}\left(0,t_{1}\right)\left[x_{t_{1}}-\Phi_{0}\left(t_{1}\right)x_{0}\right]; \end{array}$$

it follows from the solution of system (1) that

(16) x(t1)=Φ0(t1)x0+∫0t1Φ(t1−τ)Bu(τ)dτ=Φ0(t1)x0+∫0t1Φ(t1−τ)BBTΦT(t1−τ)Wc−1(0,t1)  ×[xt1−Φ0(t1)x0]dτ=Φ0(t1)x0+∫0t1Φ(t1−τ)BBTΦT(t1−τ)dτ×Wc−1(0,t1)[xt1−Φ0(t1)x0]=Φ0(t1)x0+Wc(0,t1)Wc−1(0,t1)[xt1−Φ0(t1)x0]=Φ0(t1)x0+[xt1−Φ0(t1)x0]=xt1.

Thus, the system (1) is controllable on [0, *t*_1_].

We show the converse by contradiction. Suppose that the system (1) is controllable on [0, *t*_1_], but the matrix *W*_*c*_(0, *t*_1_) is singular. Then there exists an *n* × 1 nonzero vector *v* such that

(17) 0=vTWc(0,t1)v=∫0t1vTΦ(t1−τ)BBTΦT(t1−τ)v dτ=∫0t1||vTΦ(t1−τ)B||2dτ,

which implies

(18) $$\begin{array}{c} v^{T}\Phi \left(t_{1}-\tau \right)B\equiv 0, \end{array}$$

for all *τ* ∈ [0, *t*_1_]. If (1) is controllable, there exists an input that transfers the initial *x*(0) = *x*_0_ to *x*(*t*_1_) = 0. We choose *x*_0_ = −Φ_0_^−1^(*t*_1_)*v*; then there exists an input such that

(19) $$\begin{array}{c} x\left(t_{1}\right)=-\Phi_{0}\left(t_{1}\right)\Phi_{0}^{-1}\left(t_{1}\right)v+\int_{0}^{t_{1}}\Phi \left(t_{1}-\tau \right)Bu\left(\tau \right)d\tau =0; \end{array}$$

that is,

(20) $$\begin{array}{c} v=\int_{0}^{t_{1}}\Phi \left(t_{1}-\tau \right)Bu\left(\tau \right)d\tau . \end{array}$$

Its premultiplication by *v* ^*T*^ yields

(21) $$\begin{array}{c} v^{T}v=\int_{0}^{t_{1}}v^{T}\Phi \left(t_{1}-\tau \right)Bu\left(\tau \right)d\tau =0, \end{array}$$

which contradicts *v* ≠ 0. So the matrix *W*_*c*_(0, *t*_1_) is nonsingular. The proof is thus completed.

In the following, we consider the special case of systems (1) with *A*_12_ = *A*_21_ = 0. The systems (1) are reduced to

(22) $$\begin{array}{c} \left[\begin{array}{c} {\;}^{c}D_{t}^{\alpha }x_{1}\left(t\right)\\ {\;}^{c}D_{t}^{\beta }x_{2}\left(t\right) \end{array}\right]=\left[\begin{array}{cc} A_{11} & 0\\ 0 & A_{22} \end{array}\right]\left[\begin{array}{c} x_{1}\left(t\right)\\ x_{2}\left(t\right) \end{array}\right]+\left[\begin{array}{c} B_{1}\\ B_{2} \end{array}\right]u\left(t\right), \end{array}$$

which can be rewritten as the following two subsystems:

(23a) $$\begin{array}{c} {\;}^{c}D_{t}^{\alpha }x_{1}\left(t\right)=A_{11}x_{1}\left(t\right)+B_{1}u\left(t\right), \end{array}$$

(23b) $$\begin{array}{c} {\;}^{c}D_{t}^{\beta }x_{2}\left(t\right)=A_{22}x_{2}\left(t\right)+B_{2}u\left(t\right). \end{array}$$

Thus, the following corollary is true.

**Corollary 8**

The fractional linear system (22) is controllable on [0, *t*_1_] if and only if the controllability Gramian matrix

(24) Wc(0,t1)=∫0t1[∑k=0∞(t1−τ)(k+1)α−1Γ((k+1)α)A11kB1∑l=0∞(t1−τ)(l+1)β−1Γ((l+1)β)A22lB2]  ×[∑k=0∞(t1−τ)(k+1)α−1Γ((k+1)α)A11kB1∑l=0∞(t1−τ)(l+1)β−1Γ((l+1)β)A22lB2]Tdτ

is nonsingular.

**Proof**

When *A*_12_ = *A*_21_ = 0, system (1) is reduced to the system (22). It follows from simple computation that

(25) $$\begin{array}{c} T_{kl}=\{\begin{array}{cc} I_{n},\; & k=l=0,\\ T_{01}^{l},\; & k=0,\;\;l=1,2,\ldots ,\\ T_{10}^{k},\; & l=0,\;\;k=1,2,\ldots ,\\ 0_{n},\; & \text{others}, \end{array} \end{array}$$

where

(26) T01=[000A22],  T10=[A11000],Φ0(t)=T00+T01tβΓ(β+1)+T02t2βΓ(2β+1)+⋯ +T10tαΓ(α+1)+T20t2αΓ(2α+1)+⋯=[In100In2]+tβΓ(β+1)[000A22] +t2βΓ(2β+1)[000A22]2+⋯ +tαΓ(α+1)[A11000] +t2αΓ(2α+1)[A11000]2+⋯=[∑k=0∞tkαΓ(kα+1)A11k00∑l=0∞tlβΓ(lβ+1)A22l],Φ1(t)=T00tα−1Γ(α)+T01tα+β−1Γ(α+β)+T02tα+2β−1γ(α+2β)+⋯ +T10t2α−1Γ(2α)+T20t3α−1Γ(3α)+⋯=tα−1Γ(α)[In100In2]+tα+β−1Γ(α+β)[000A22] +tα+2β−1Γ(α+2β)[000A22]2+⋯ +t2α−1Γ(2α)[A11000] +t3α−1Γ(3α)[A11000]2+⋯=[∑k=0∞t(k+1)α−1Γ((k+1)α)A11k00∑l=0∞tα+lβ−1Γ(α+lβ)A22l],Φ2(t)=T00tβ−1Γ(β)+T01t2β−1Γ(2β)+T02t3β−1Γ(3β)+⋯ +T10tα+β−1Γ(α+β)+T20t2α+β−1Γ(2α+β)+⋯=tβ−1Γ(β)[In100In2]+t2β−1Γ(2β)[000A22] +t3β−1Γ(3β)[000A22]2+⋯ +tα+β−1Γ(α+β)[A11000]+t2α+β−1Γ(2α+β)[A11000]2+⋯=[∑k=0∞tkα+β−1Γ(kα+β)A11k00∑l=0∞t(l+1)β−1Γ((l+1)β)A22l].

Therefore, the controllability Gramian matrix in the Theorem 7 is reduced to

(27) Wc(0,t1)=∫0t1[∑k=0∞(t1−τ)(k+1)α−1Γ((k+1)α)A11kB1∑l=0∞(t1−τ)(l+1)β−1Γ((l+1)β)A22lB2]  ×[∑k=0∞(t1−τ)(k+1)α−1Γ((k+1)α)A11kB1∑l=0∞(t1−τ)(l+1)β−1Γ((l+1)β)A22lB2]Tdτ.

This completes the proof.

Obviously, the following proposition is true.

**Proposition 9**

The fractional linear system (22) is controllable if and only if subsystems (23a) and (23b) are all controllable.

In the following, we consider another special case of system (1). When *α* = *β* in the system (1), it is reduced to

(28) $$\begin{array}{c} {\;}^{c}D_{t}^{\alpha }x\left(t\right)=Ax\left(t\right)+Bu\left(t\right), \end{array}$$

where

(29) $$\begin{array}{c} x\left(t\right)=\left[\begin{array}{c} x_{1}\left(t\right)\\ x_{2}\left(t\right) \end{array}\right],\;\;A=\left[\begin{array}{cc} A_{11} & A_{12}\\ A_{21} & A_{22} \end{array}\right],\;\;B=\left[\begin{array}{c} B_{1}\\ B_{2} \end{array}\right]. \end{array}$$

**Corollary 10**

The fractional linear system (28) is controllable on [0, *t*_1_] if and only if the controllability Gramian matrix

(30) Wc=∫0t1∑k=0∞(t1−τ)(k+1)α−1Γ((k+1)α)AkBBT  ×∑K=0∞(t1−τ)(k+1)α−1Γ((k+1)α)(AT)kdτ

is nonsingular.

**Proof**

According to the result of Lemma 5, when *α* = *β*, we can obtain

(31) Φ0(t)=∑k=0∞(Tk0tkαΓ(kα+1)+Tk1t(k+1)αΓ((k+1)α+1)+⋯)=T00+T01tαΓ(α+1)+T02t2αΓ(2α+1) +T03t3αΓ(3α+1)+⋯+T10tαΓ(α+1) +T11t2αΓ(2α+1)+T12t3αΓ(3α+1)+⋯ +T20t2αΓ(2α+1)+T21t3αΓ(3α+1) +T22t4αΓ(4α+1)+⋯+T30t3αΓ(3α+1) +T31t4αΓ(4α+1)+T32t5αΓ(5α+1)+⋯ ⋯=T00+tαΓ(α+1)(T01+T10) +t2αΓ(2α+1)(T02+T11+T20) +t3αΓ(3α+1)(T03+T12+T21+T30)+⋯=[In100In2]+tαΓ(α+1)A+t2αΓ(2α+1)A2 +t3αΓ(3α+1)A3+⋯=∑k=0∞tkαΓ(kα+1)Ak.

For the same reason as before, we get

(32) Φ1(t)=Φ2(t)=∑k=0∞ ∑l=0∞Tklt(k+l+1)α−1Γ((k+l+1)α)=∑k=0∞t(k+1)α−1Γ((k+1)α)Ak.

Therefore, by simple computation, the controllability Gramian matrix of the system (28) can be obtained as (30). The proof is thus completed.

**Remark 11**

Corollary 10 is equivalent to the result of Theorem  2.2 in [21]. Therefore, Theorem 7 of this paper extends the existing results to a more general case.

## 4. Observability

In this section, we treat another fundamental property of the fractional linear system with different orders, namely, observability with respect to a linear output. Throughout the rest of this paper, we consider the system (1) with the following output equation:

(33) $$\begin{array}{c} \left[\begin{array}{c} y_{1}\left(t\right)\\ y_{2}\left(t\right) \end{array}\right]=\left[\begin{array}{cc} C_{11} & C_{12}\\ C_{21} & C_{22} \end{array}\right]\left[\begin{array}{c} x_{1}\left(t\right)\\ x_{2}\left(t\right) \end{array}\right], \end{array}$$

where *y*_1_ ∈ *R* ^*p*_1_^,  *y*_2_ ∈ *R* ^*p*_2_^ are the output vectors; *x*_1_ ∈ *R* ^*n*_1_^,  *x*_2_ ∈ *R* ^*n*_2_^ are the state vectors in the system (1); *C*_*ij*_ ∈ *R* ^*p*_*i*_×*n*_*j*_^,  *i*, *j* = 1,2, are the known constant matrices; *p*_1_ + *p*_2_ = *p*.

When *C*_12_ = *C*_21_ = 0, the output (33) is reduced to the following simple form:

(34) $$\begin{array}{c} \left[\begin{array}{c} y_{1}\left(t\right)\\ y_{2}\left(t\right) \end{array}\right]=\left[\begin{array}{cc} C_{11} & 0\\ 0 & C_{22} \end{array}\right]\left[\begin{array}{c} x_{1}\left(t\right)\\ x_{2}\left(t\right) \end{array}\right], \end{array}$$

which is equivalent to two suboutput equations as follows:

(35a) $$\begin{array}{c} y_{1}=C_{11}x_{1}, \end{array}$$

(35b) $$\begin{array}{c} y_{2}=C_{22}x_{2}. \end{array}$$

**Definition 12**

The system (1) with the output (33) are called state observable on [0, *T*] if any initial state *x*(0) = *x*_0_ ∈ *R* ^*n*^ can be uniquely determined by the corresponding system input *u*(*t*) and system output *y*(*t*), for *t* ∈ [0, *T*].

Define observability Gramian matrix *W*_*o*_(0, *t*) as

(36) $$\begin{array}{c} W_{o}\left(0,t\right)=\int_{0}^{t}\Phi_{0}^{T}\left(\tau \right)C^{T}C\Phi_{0}\left(\tau \right)d\tau , \end{array}$$

where

(37) $$\begin{array}{c} C=\left[\begin{array}{cc} C_{11} & C_{12}\\ C_{21} & C_{22} \end{array}\right]. \end{array}$$

**Theorem 13**

The system (1) with the output (33) is observable on [0, *t*_1_] if and only if the observability Gramian matrix

(38) $$\begin{array}{c} W_{o}\left(0,t_{1}\right)=\int_{0}^{t_{1}}\Phi_{0}^{T}\left(\tau \right)C^{T}C\Phi_{0}\left(\tau \right)d\tau \end{array}$$

is invertible.

**Proof**

It follows from Lemma 4 that the output of system (1) has the following expression:

(39) y(t)=Cx(t)=CΦ0(t)x0+C∫0tΦ(t−τ)Bu(τ)dτ.

It is easy to see from Definition 12 that the observability of system (1) is equivalent to the observability of *y*(*t*) given by

(40) $$\begin{array}{c} y\left(t\right)=C\Phi_{0}\left(t\right)x_{0}, \end{array}$$

as *u*(*t*) = 0.

Multiplying both sides of (40) by Φ_0_^*T*^(*t*)*C* ^*T*^, and integrating with respect to *t* from 0 to *t*_1_, we have

(41) $$\begin{array}{c} \int_{0}^{t_{1}}\Phi_{0}^{T}\left(t\right)C^{T}y\left(t\right)dt=W_{o}\left(0,t_{1}\right)x_{0}. \end{array}$$

Obviously, the left-hand side of (41) depends on *y*(*t*), and the right-hand side in (41) does not depend on *y*(*t*), *t* ∈ [0, *t*_1_]. Thus, (41) is a linear algebraic equation of *x*_0_. If *W*_*o*_(0, *t*_1_) is invertible, then the initial state *x*(0) = *x*_0_ is uniquely determined by the corresponding system output *y*(*t*), for *t* ∈ [0, *t*_1_]. Namely, the system (1) is observable on [0, *t*_1_].

Next we show that if *W*_*o*_(0, *t*_1_) is singular for all *t*_1_, then system (1) with the output (33) is not observable. Suppose *W*_*o*_(0, *t*_1_) is singular; then there exists an *n* × 1 nonzero constant vector *v* such that

(42) vTWo(0,t1)v=∫0t1vTΦ0T(τ)CTCΦ0(τ)v dτ=∫0t1||CΦ0(τ)v||2dτ=0,

which implies that

(43) $$\begin{array}{c} C\Phi_{0}\left(\tau \right)v\equiv 0, \end{array}$$

for all *τ* ∈ [0, *t*_1_]. If we choose *x*(0) = *x*_0_ = *v*, then the output (33) is given by

(44) $$\begin{array}{c} y\left(t\right)=C\Phi_{0}\left(t\right)x_{0}=C\Phi_{0}\left(t\right)v\equiv 0. \end{array}$$

Thus, the initial state *x*_0_ cannot be uniquely determined by *y*(*t*). Therefore, the system (1) with the output (33) is not observable. This completes the proof.

**Remark 14**

When *α* = *β* in the system (1), Φ_0_(*t*) is already obtained in the proof of Corollary 10. Therefore, the observability Gramian matrix in Theorem 13 is

(45) Wo(0,t1)=∫0t1Φ0T(τ)CTCΦ0(τ)dτ=∫0t1∑k=0∞τkαΓ(kα+1)(AT)kCTC∑k=0∞τkαΓ(kα+1)Akdτ,

which is the observability Gramian matrix in paper [22] by denoting *E*_*α*_(*At* ^*α*^) = ∑_*k*=0_^*∞*^(*τ* ^*kα*^/Γ(*kα* + 1))*A* ^*k*^. Therefore, Theorem 13 is actually a generalization of the existing observability results for the fractional linear system.

When *A*_12_ = *A*_21_ = 0 and *C*_12_ = *C*_21_ = 0, the system (1) with the output (33) is reduced to the following state equation and output equation:

(46a) $$\begin{array}{c} \left[\begin{array}{c} {}^{c}D_{t}^{\alpha }x_{1}\left(t\right)\\ {}^{c}D_{t}^{\beta }x_{2}\left(t\right) \end{array}\right]=\left[\begin{array}{cc} A_{11} & 0\\ 0 & A_{22} \end{array}\right]\left[\begin{array}{c} x_{1}\left(t\right)\\ x_{2}\left(t\right) \end{array}\right]+\left[\begin{array}{c} B_{1}\\ B_{2} \end{array}\right]u\left(t\right), \end{array}$$

(46b) $$\begin{array}{c} \left[\begin{array}{c} y_{1}\left(t\right)\\ y_{2}\left(t\right) \end{array}\right]=\left[\begin{array}{cc} C_{11} & 0\\ 0 & C_{22} \end{array}\right]\left[\begin{array}{c} x_{1}\left(t\right)\\ x_{2}\left(t\right) \end{array}\right], \end{array}$$

which can be rewritten as the following two subsystems with input and output:

(47a) $$\begin{array}{c} {\;}^{c}D_{t}^{\alpha }x_{1}\left(t\right)=A_{11}x_{1}\left(t\right)+B_{1}u\left(t\right), \end{array}$$

(47b) $$\begin{array}{c} y_{1}\left(t\right)=C_{11}x_{1}\left(t\right), \end{array}$$

(48a) $$\begin{array}{c} {\;}^{c}D_{t}^{\beta }x_{1}\left(t\right)=A_{22}x_{2}\left(t\right)+B_{2}u\left(t\right), \end{array}$$

(48b) $$\begin{array}{c} y_{2}\left(t\right)=C_{22}x_{2}\left(t\right). \end{array}$$

It follows from conditions *A*_12_ = *A*_21_ = 0 and *C*_12_ = *C*_21_ = 0 that

(49) Φ0T(t)CTCΦ0(t)=[∑k=0∞tkαΓ(kα+1)A11k00∑l=0∞tlβΓ(lβ+1)A22l]T[C1100C22]T[C1100C22][∑k=0∞tkαΓ(kα+1)A11k00∑l=0∞tlβΓ(lβ+1)A22l]=[∑k=0∞tkαΓ(kα+1)(A11k)T×C11TC11×∑k=0∞tkαΓ(kα+1)A11k00∑l=0∞tlβΓ(lβ+1)(A22l)T×C22TC22×∑l=0∞tlβΓ(lβ+1)A22l]=[Eα(A11Ttα)C11T×C11Eα(A11tα)00Eβ(A22Ttβ)C22T×C22Eβ(A22tβ)],

where *E*_*s*_(*At* ^*s*^) = ∑_*k*=0_^*∞*^(*t* ^*ks*^/Γ(*ks* + 1))  *A* ^*k*^ is Mittag-Leffler function.

Therefore, the following corollary holds.

**Corollary 15**

Denote *E*_*α*_(*A*_11_, *t*) = *E*_*α*_(*A*_11_^*T*^ *t* ^*α*^)*C*_11_^*T*^ × *C*_11_ *E*_*α*_(*A*_11_ *t* ^*α*^) and *E*_*β*_(*A*_22_, *t*) = *E*_*β*_(*A*_22_^*T*^ *t* ^*β*^)*C*_22_^*T*^ × *C*_22_ *E*_*β*_(*A*_22_ *t* ^*β*^). Then the system (46a) with the output (46b) is observable on [0, *t*_1_] if and only if the observability Gramian matrix

(50) $$\begin{array}{c} \int_{0}^{t_{1}}\left[\begin{array}{cc} E_{\alpha }\left(A_{11},t\right) & 0\\ 0 & E_{\beta }\left(A_{22},t\right) \end{array}\right]dt \end{array}$$

is nonsingular.

The following proposition is also true.

**Proposition 16**

The fractional linear system (46a) with the output (46b) is observable if and only if the fractional linear subsystems (47a) with the output (47b) and (48a) with the output (48b) are all observable.

## 5. Conclusions

In this paper, the controllability and observability problems for fractional linear systems with two different orders have been studied. The sufficient and necessary conditions for state controllability and state observability of such systems are established. The results obtained will be useful in the analysis and synthesis of fractional dynamical systems. Extending the results of this paper toward fractional linear systems consisting of *n* subsystems with different orders is a future work.
